# The role of kurtosis and kurtosis-adjusted energy metric in occupational noise-induced hearing loss among metal manufacturing workers

**DOI:** 10.3389/fpubh.2023.1159348

**Published:** 2023-06-29

**Authors:** Shuangyan Liu, Hua Zou, Song Lei, Jiarui Xin, Peiyi Qian, Yun Liu, Yingqi Chen, Kuai Yu, Meibian Zhang

**Affiliations:** ^1^School of Public Health, Tongji Medical College, Huazhong University of Science and Technology, Wuhan, Hubei, China; ^2^Institute of Occupational Health and Radiation Protection, Zhejiang Provincial Center for Disease Control and Prevention, Hangzhou, Zhejiang, China; ^3^Ningbo Center for Disease Control and Prevention, Ningbo, Zhejiang, China; ^4^Chinese Center for Disease Control and Prevention, Beijing, China; ^5^School of Public Health, Hangzhou Normal University, Hangzhou, Zhejiang, China

**Keywords:** occupational health, occupational noise, hearing loss, kurtosis, cumulative noise exposure

## Abstract

**Background:**

Noise energy has been well-established to increase the risk of occupational noise-induced hearing loss (NIHL). However, the role of noise temporal structure (expressed by kurtosis) or its combination with energy metrics (e.g., kurtosis-adjusted cumulative noise exposure, adj-CNE) in occupational NIHL was still unclear.

**Methods:**

A cross-sectional survey of 867 Chinese workers, including 678 metal manufacturing workers and 189 workers exposed to Gaussian noise, was conducted. Noise energy metrics, including L_Aeq,8h_ and CNE, kurtosis (β), and adj-CNE were used to quantify noise exposure levels. Noise-induced permanent threshold shift at frequencies 3, 4, and 6 kHz (NIPTS_346_) and the prevalence of high-frequency NIHL (HFNIHL%) were calculated for each participant. The dose–response relationship between kurtosis or adj-CNE and occupational NIHL was observed.

**Results:**

Among 867 workers, different types of work had specific and independent noise energy and kurtosis values (*p* > 0.05). HFNIHL% increased with an increase in exposure duration (ED), L_Aeq,8h_, CNE, or kurtosis (*p* < 0.01), and there were strong linear relationships between HFNIHL% and ED (coefficient of determination [*R*^2^] = 0.963), CNE (*R*^2^ = 0.976), or kurtosis (*R*^2^ = 0.938, when CNE < 100 dB(A)∙year). The “V” shape notching extent in NIPTS became deeper with increasing kurtosis when CNE < 100 dB(A)∙year and reached the notching bottom at the frequency of 4 or 6 kHz. The workers exposed to complex noise (β ≥ 10) had a higher risk of NIHL than those exposed to Gaussian noise (β < 10) at the frequencies of 3, 4, 6, and 8 kHz (OR > 2, *p* < 0.01). Moreover, HFNIHL% increased with adj-CNE (*p* < 0.001). There were strong linear relationships between NIHL and adj-CNE or CNE when β ≥ 10 (*R*^2^_adj-CNE_ > *R*^2^_CNE_). After CNE was adjusted by kurtosis, average differences in NIPTS_346_ or HFNIHL% between the complex and Gaussian noise group were significantly reduced (*p* < 0.05).

**Conclusion:**

Kurtosis was a key factor influencing occupational NIHL among metal manufacturing workers, and its combination with energy metrics could assess the risk of NIHL more effectively than CNE alone.

## Introduction

Noise-induced hearing loss (NIHL) caused by accumulated noise exposure, second only to senile deafness in terms of the incidence (1), is one of the major types of sensorineural hearing loss. Worldwide, approximately 5% of the population suffers from NIHL (2). It was estimated that approximately 600 million workers were exposed to harmful noise levels (3), and about 16% of disabling hearing loss in adults could be directly attributed to occupational noise exposure (4). As a major occupational health risk, NIHL has been a global public health problem (5, 6). In the US, NIHL is the most common occupational disease (7). In China, occupational NIHL is the second major occupational disease (8). The prevalence of NIHL (NIHL%) in the transportation and manufacturing industries is between 18 and 67% in low/middle-income countries (9, 10).

The current widely-accepted model of NIHL (ISO-1999, 2013) was established and implemented based on the equal-energy hypothesis (EEH), which assumes that the damage to the auditory system caused by noise exposure is proportional to the duration of exposure multiplied by the noise intensity. That is to say it is independent of the risk of hearing loss and noise’s temporal characteristics. However, in real occupational environments, complex non-Gaussian noise with impulsive components is ubiquitous, and it is composed of a transient high-energy impulsive noise superimposed on stationary (Gaussian) background noise (11). Animal experiments and epidemiological studies suggested that the EEH was not appropriate for the risk assessment of complex noise, and that it might underestimate the effect of occupational complex noise on the risk of NIHL (12–15). Lempert et al. observed the accuracy of ISO-1999 in the risk assessment of NIHL using previous data. They found that ISO-1999 closely predicted neither the distribution of hearing threshold levels nor the database of the National Institute for Occupational Safety and Health, indicating that lower estimates of the risk of NIHL were found using ISO-1999 (16). Despite having the same equivalent sound levels, the damage to the auditory system caused by complex noise was more serious than that caused by Gaussian noise (17). These findings suggest that the role of noise temporal structure in NIHL caused by complex noise should be addressed.

Kurtosis (β), which indirectly reflects the temporal structure of noise exposure, was proposed by Erdreich (18). Kurtosis is the ratio of the fourth-order central moment to the squared second-order central moment of a distribution. It is a statistical metric that simplifies some time-domain variables of noise (e.g., pulse peak value, duration, and inter-pulse distribution) that affect hearing, and is easy to calculate. Animal studies initially showed that besides noise energy, noise kurtosis plays an important role in NIHL development (19, 20). Subsequently, some scholars began to apply kurtosis-adjusted noise energy to assess occupational NIHL among workers. The dose–response relationship between the NIHL% and kurtosis-adjusted cumulative noise exposure (adj-CNE) among workers exposed to complex noise and the validity of adj-CNE have been found in some industries, such as the steel manufacturing, metal fabrication, furniture, automobile, and general equipment manufacturing industries (14, 15, 21, 22). However, these findings on the role of the adj-CNE metric in occupational NIHL need to be validated in a broader range of specific industries.

Herein, a cross-sectional study was designed to validate the role of kurtosis and adj-CNE in occupational NIHL among metal manufacturing workers. These workers exposed to complex noise were selected as subjects, and workers exposed to Gaussian noise from the paper-making and textile industries served as controls. Whether the dose–response relationship between adj-CNE and NIHL was closer to that of Gaussian noise served as a criterion to judge the effectiveness of adj-CNE.

## Materials and methods

### Subjects

A total of 867 Chinese workers, including 678 metal manufacturing workers and 189 workers exposed to Gaussian noise from the paper-making and textile industries in Zhejiang Province of East China, were continuously selected during 2017 and 2018 for the cross-sectional survey. The inclusion criteria for the subjects were presented in Supplementary Table S1.

### Questionnaire survey

According to the needs of the investigation, a uniform questionnaire was designed for each subject. The collected data were shown in Supplementary Table S2. Subjects were interviewed to complete questionnaires by trained investigators and were asked to sign an informed consent form. The study was approved by the ethics committee of the Zhejiang Center for Disease Control and Prevention, China (ZJCDC-T-043-R). All methods were performed following relevant guidelines and regulations.

### Noise waveform recording and analysis

The noise exposure data for subjects for the entire shift duration were collected using a digital noise dosimeter (ASV5910-R, Hangzhou Aihua Instrument Co., Ltd.), which can operate continuously at a sampling rate of 48 kHz. The digital noise dosimeter was equipped with a 1/4-inch microphone with a 10–20 kHz frequency response range and a 40–141 dB(A) measurement range. An equivalent continuous A-weighted noise exposure level normalized to an 8-h working day (L_Aeq,8h_) can be measured using the noise dosimeter, which was attached to the participants’ clothing at the shoulder by clips, with the microphone pointing up. After recording noise exposure data, the data were transmitted from the recorder to a computer for subsequent analysis. The noise dosimeter was calibrated using a sound level calibrator (Hangzhou Aihua Instrument, AWA6221B) before and after each sampling cycle. MATLAB software (Natick, MA) calculated the sampling kurtosis by analyzing the shift-long noise. The equation for calculating kurtosis is shown in Formula (1).


(1) $$\beta =\frac{m_{4}}{m_{2}}=\frac{\frac{1}{n}\sum_{i=1}^{n}{\left(x_{i}-\bar{x}\right)}^{4}}{{\left(\frac{1}{n}\sum_{i=1}^{n}{\left(x_{i}-\bar{x}\right)}^{2}\right)}^{2}}$$

$x_{i}$ is the *i*th value, $\bar{x}$ is the sample mean, and β is noise kurtosis. Theoretically, the kurtosis value of Gaussian noise is 3 (*β* = 3) and that of complex non-Gaussian noise is greater than 3. A 40-s window is acceptable for kurtosis measurement based on previous animal data using a similar 48 kHz sampling rate (20, 23). The mean kurtosis calculated in a 40-s window was used as the kurtosis value of the entire shift time. In this study, a mean kurtosis of 10 was used as the boundary value between Gaussian and complex noise (12, 19, 24). Noise with a mean *β* < 10 was defined as continuous Gaussian noise, and that with a mean β ≥ 10 was defined as complex noise. The greater the kurtosis, the higher the impulsiveness of the complex noise (25).

A comprehensive noise exposure metric (i.e., cumulative noise exposure, CNE), including L_Aeq,8h_ and the ED, was used to quantify noise energy for each subject according to Formula (2) (14). By incorporating time-domain variables into the evaluation of complex noise environments and unifying CNE calculations for epidemiological data, including both Gaussian and complex noise, the adj-CNE was calculated according to Formula (3) (14).


(2) $$\text{CNE}=L_{\text{Aeq},8h}+10\text{logT}$$

(3) $$\text{adj}-{\text{CNE}}_{\text{Kurtosis}-\text{adjusted}}=L_{\text{Aeq},8h}+\frac{\ln\left(\beta \right)+1.9}{\log\left(2\right)}\text{logT}$$

*T* is the ED in years. L_Aeq,8h_ is measured in dB(A). The units of CNE and adj-CNE are both dB(A)∙year. When Gaussian noise has a kurtosis of β = 3, the term [$\frac{\ln\left(\beta \right)+1.9}{\log\left(2\right)}$] becomes equal to 10. Thus, for Gaussian noise, the adj-CNE equals the unadjusted CNE. Equation (3) shows that when L_Aeq,8h_ is fixed, the adj-CNE will be larger for complex noise (β ≥ 10) than for Gaussian noise (β < 10).

### Hearing testing and hearing loss diagnosis

Pure-tone audiometry was performed for each participant in a sound-insulation room by experienced audiologists. The test was conducted on the left and right ears at 0.5, 1.0, 2.0, 3.0, 4.0, 6.0, and 8.0 kHz. All subjects were required to be outside their daily noise environment for at least 16 h before the test. Because the worker population in this study was rigorously screened, the pure-tone hearing threshold levels (HTLs) were adjusted according to sex and age by following Annex A, Table A.3, of ISO 1999:2013. In this study, high-frequency NIHL (HFNIHL) was diagnosed as one or more adjusted HTLs, in either ear, at 3, 4, or 6 kHz equal to or higher than 30 dB based on an extensive body of prior research (14, 15, 21, 22, 26, 27). The analysis focused on the noise-sensitive frequency range of 3, 4, and 6 kHz because the noise-induced hearing loss from continuous noise occurs predominantly in this range initially (28). The noise-induced permanent threshold shift at 3.0, 4.0, and 6.0 kHz (NIPTS_346_) was calculated according to the Annex A of ISO 1999:2013.

### Statistical analysis

Continuous variables with normal distribution were expressed as mean ± standard deviation (SD), and categorical variables were presented as percentages. The correlations between kurtosis and L_Aeq,8h_ or CNE were described using a scatter diagram and correlation coefficient (r). Considering the effects of work types on kurtosis and L_Aeq,8h_, the partial correlation coefficient between kurtosis and L_Aeq,8h_ was calculated after adjusting for work types. The cut-off points for ED (1, 5, 10, 15), CNE (80, 100, 110, 120) and kurtosis (10, 50, 100, 200) were determined based on our previous studies (21, 22). The dose–response relationship between different noise exposure metrics (e.g., L_Aeq,8h_, ED, CNE, kurtosis, and adj-CNE) and the prevalence of HFNIHL (HFNIHL%) was observed by the Cochran-Armitage trend chi-squared test.

The linear and nonlinear relationships between exposure metrics (e.g., ED, CNE, and kurtosis) and HFNIHL% were evaluated using the regression equation, including linear, logarithmic, inverse, quadratic, cubic, composite, power, sigmoid curve, growth, and exponential models. The cut-off value for age was determined based on the receiver operating characteristic (ROC) curve. Subjects were divided into two groups, with an age of 33 years set as the cut-off value. Logistic regression analysis was used to evaluate the role of kurtosis in NIHL% at each frequency, and the effect was presented as odds ratios (OR) with 95% confidence intervals (CI). The differences in NIPTS_346_ and HFNIHL% between complex noise exposure workers and Gaussian noise exposure workers before and after CNE adjustment by kurtosis were evaluated using paired sample t-test. CNE was further divided into eight subgroups based on the cut-off points of 90, 95, 100, 105, 110, 115, and 120 as shown in our previous studies (21, 22, 27). Herein, the dose–response relationship of Gaussian noise served as a baseline to determine whether adj-CNE could effectively assess NIHL. When *p* < 0.05, the difference was considered statistically significant.

## Results

### Noise exposure characteristics and NIHL among manufacturing workers

**Figure 1:**
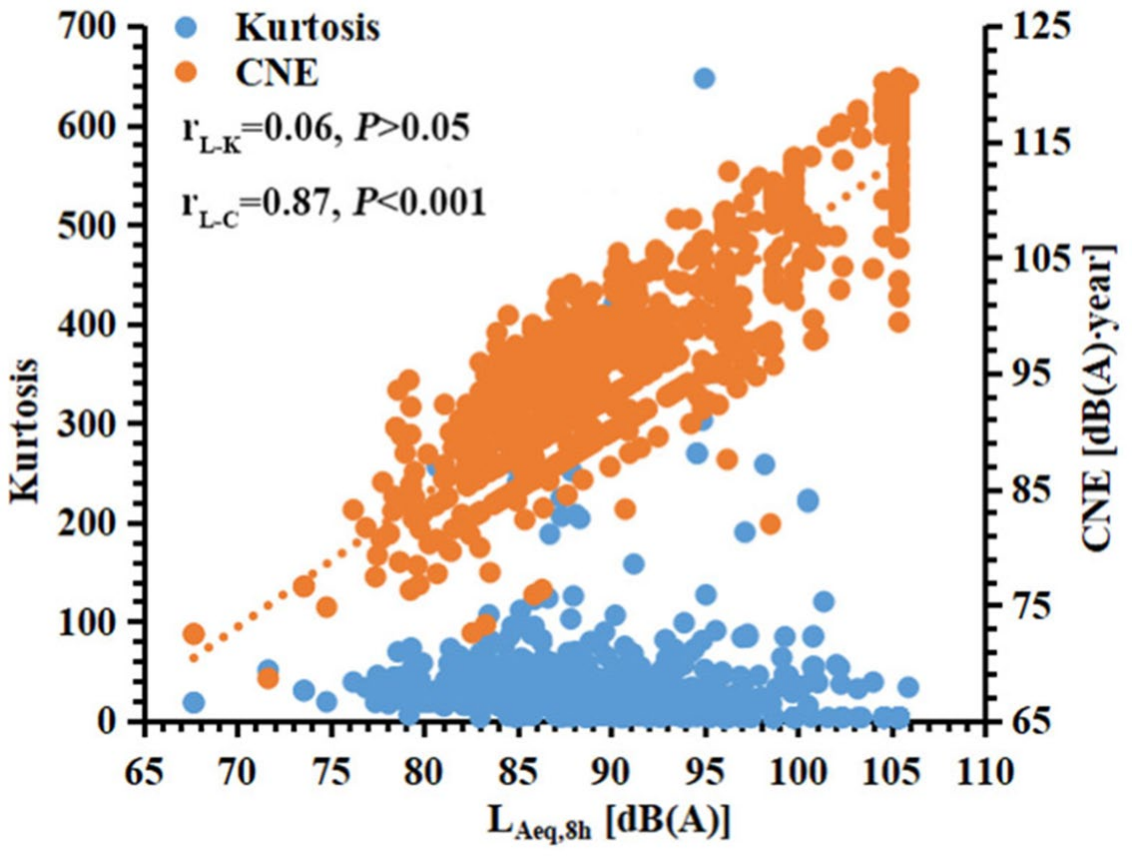
The correlation between kurtosis or CNE and L_Aeq,8h_. r_L-K_ is the partial correlation coefficient between L_Aeq,8h_ and kurtosis; r_L-C_ is the linear correlation coefficient between L_Aeq,8h_ and CNE.

Table 1 shows the general information, ED, noise energy, and kurtosis among 867 manufacturing workers. Overall, the average age and ED were 35.61 ± 9.45 years and 6.88 ± 6.82 years, with 59.52% of males. The majority of workers were exposed to high noise levels (> 85 dB(A)) and complex noise (β ≥ 10). Among the 678 metal manufacturing workers, the mean L_Aeq,8h_, CNE, kurtosis, and NIPTS_346_ were 88.02 ± 5.24 dB(A), 93.00 ± 6.71 dB(A)∙year, 40.91 ± 51.22, and 26.60 ± 15.62 dB HL, respectively, with 40.41% of HFNIHL. In detail, forging, grinding, or polishing workers had the highest NIPTS_346_ (37.23 ± 14.95 dB HL) and the highest HFNIHL% (80.65%). Figure 1 illustrates a positive correlation between L_Aeq,8h_ and CNE (r_L-C_ = 0.87, *p <* 0.001), while no significant correlation between kurtosis and L_Aeq,8h_ (r_L-K_ = 0.06, *p* > 0.05) was found.

**Table 1:** General information, noise exposure, NIPTS_346_ and HFNIHL% for workers from typical work types.

Industries	Work types	*N*	Age, Mean ± SD, year	Male/ female, n	ED Mean ± SD, year	L_Aeq.8h_, dB(A)		CNE, dB(A)∙year		Kurtosis	Adj-CNE, dB(A)∙year		NIPTS_346_, Mean ± SD, dB	HFNIHL (%)
						Mean ± SD	≥85 (%)	Mean ± SD	≥100 (%)	Mean ± SD	Mean ± SD	≥100 (%)		
Paper/textile industries	Pulping	35	44.66 ± 11.65	21/14	12.89 ± 9.81	90.17 ± 4.08	91.40	99.78 ± 4.98	54.30	4.55 ± 0.85	99.78 ± 4.98	54.30	25.25 ± 11.90	40.00
	Spinning	154	31.3 ± 8.52	75/79	12.29 ± 8.22	101.23 ± 4.19	99.35	110.60 ± 6.10	94.16	3.30 ± 0.00	110.60 ± 6.10	94.16	27.24 ± 14.14	47.40
	Total	189	33.77 ± 10.52	96/93	12.40 ± 8.51	99.18 ± 5.99	97.88	108.60 ± 7.24	86.77	3.53 ± 0.60	108.60 ± 7.24	86.77	26.87 ± 13.74	46.03
Metal manufacturing industry	Assembling	192	33.91 ± 8.15	73/119	5.60 ± 5.15	85.90 ± 4.20	54.69	91.36 ± 6.35	4.69	46.34 ± 46.39	95.88 ± 9.65	35.42	22.52 ± 11.85	29.17
	Cold heading	53	34.85 ± 9.92	50/3	5.21 ± 4.99	88.33 ± 2.21	92.45	93.86 ± 4.96	9.43	23.17 ± 12.76	97.46 ± 7.38	30.19	28.50 ± 13.75	41.51
	Machining	83	30.31 ± 7.20	63/20	2.05 ± 3.41	89.27 ± 5.86	80.72	90.76 ± 6.29	6.02	55.59 ± 91.11	92.22 ± 7.79	12.05	26.11 ± 13.88	39.76
	Sorting, or packing	104	36.63 ± 7.22	47/57	5.67 ± 4.84	85.70 ± 3.89	54.81	91.95 ± 5.79	5.77	27.61 ± 12.47	96.46 ± 7.95	33.65	24.67 ± 11.69	35.58
	Welding	55	40.02 ± 9.53	40/15	6.24 ± 4.80	90.16 ± 4.55	92.73	95.46 ± 6.06	20.00	63.51 ± 69.29	102.45 ± 9.09	54.55	31.42 ± 16.79	50.91
	Silk making	54	37.56 ± 9.19	42/12	4.46 ± 5.31	87.04 ± 4.45	83.33	91.33 ± 6.14	7.41	24.67 ± 13.51	94.35 ± 8.65	27.78	24.76 ± 13.48	38.89
	Forging, or grinding, or polishing	31	44.00 ± 6.58	22/9	11.16 ± 9.72	91.97 ± 7.33	83.87	99.58 ± 10.41	54.84	49.54 ± 58.26	107.09 ± 13.78	58.06	37.23 ± 14.95	80.65
	Punching, or stamping	35	41.00 ± 8.15	17/18	4.17 ± 4.36	95.67 ± 3.81	100.00	99.36 ± 5.63	40.00	26.14 ± 13.34	103.02 ± 7.08	65.71	32.26 ± 18.18	60.00
	Chamfering, or turning lathe	24	35.21 ± 8.69	24/23	6.77 ± 4.56	87.17 ± 5.07	54.17	93.10 ± 3.99	0.00	27.42 ± 16.81	98.80 ± 5.30	45.83	19.22 ± 11.04	25.00
	Heat treating	24	39.33 ± 9.88	24/0	5.68 ± 3.63	87.37 ± 3.25	87.50	93.76 ± 4.99	8.33	21.64 ± 5.96	97.96 ± 6.99	50.00	31.28 ± 17.23	60.00
	Others	23	43.22 ± 10.27	19/4	5.91 ± 4.34	92.70 ± 5.10	86.96	96.14 ± 6.72	26.09	72.05 ± 76.66	104.65 ± 8.13	65.22	39.40 ± 21.68	69.57
	Total	678	36.13 ± 9.07	420/258	5.34 ± 5.34	88.02 ± 5.24	72.12	93.00 ± 6.71	11.65	40.91 ± 51.22	97.41 ± 9.54	37.32	26.60 ± 15.62	40.41
Total	**–**	867	35.61 ± 9.45	516/351	6.88 ± 6.82	90.45 ± 7.12	77.74	96.40 ± 9.39	28.03	32.76 ± 47.85	99.85 ± 10.20	48.10	26.67 ± 14.43	41.64

ED, exposure duration; NIPTS_346_, noise-induced permanent threshold shift at 3, 4, and 6 kHz frequencies; HFNIHL, high-frequency noise-induced hearing loss.

### The role of noise energy and kurtosis in the risk of occupational NIHL

**Figure 2:**
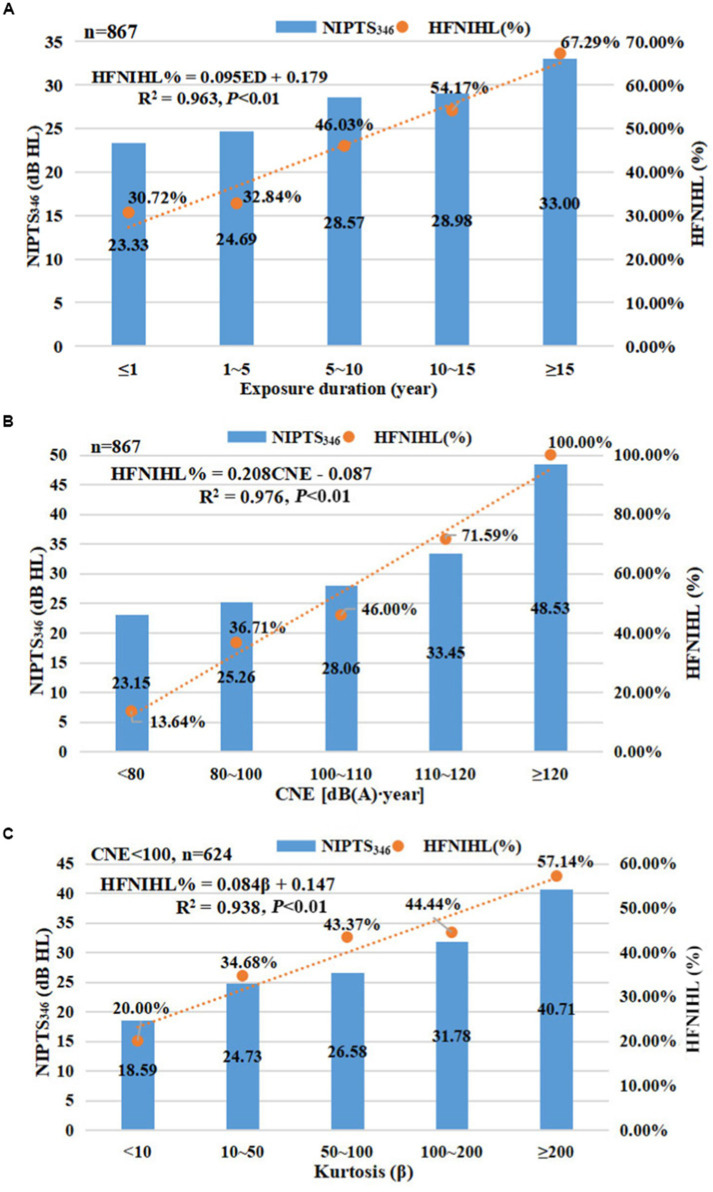
The linear relationship between ED, CNE, or kurtosis and HFNIHL%. **(A)** There was a strong linear relationship between ED and HFNIHL%, and the linear regression equations was: HFNIHL% = 0.095 ED + 0.179 (*R*^2^ = 0.963); **(B)** There was a strong linear relationship between CNE and HFNIHL%, and the linear regression equations was: HFNIHL% = 0.208 CNE + 0.087 (*R*^2^ = 0.976); **(C)** There was a strong linear relationship between kurtosis and HFNIHL% when CNE < 100 dB(A).year, and the linear regression equations was: HFNIHL% = 0.084 β + 0.147 (*R*^2^ = 0.938). ED: exposure duration; NIPTS_346_: noise-induced permanent threshold shift at 3, 4, and 6 kHz frequencies; HFNIHL: high-frequency noise-induced hearing loss.

Trend chi-squared test results showed (Table 2) the relationship between HFNIHL% (or NIPTS_346_) and noise energy metrics (ED, L_Aeq,8h_, and CNE) and kurtosis metric. The mean NIPTS_346_ and HFNIHL% increased with increasing ED, L_Aeq,8h_, and CNE (*p* < 0.001). NIPTS_346_ and HFNIHL% in the complex noise exposure group (β ≥ 10) were much higher than those in the Gaussian noise exposure group (β < 10), and the group with β ≥ 200 had the highest NIPTS_346_ (40.71 ± 26.87 dB HL) and HFNIHL% (57.14%). HFNIHL% increased with an increase in kurtosis value when CNE < 100 dB(A)∙year (*p* < 0.01). Moreover, Figures 2A–C show a strong linear relationship between HFNIHL% and ED, CNE, or kurtosis; the linear regression equations were: HFNIHL% = 0.095 ED + 0.179 (coefficient of determination [*R*^2^] = 0.963, *p* < 0.01), HFNIHL% = 0.208 CNE - 0.087 (*R*^2^ = 0.976, *p* < 0.01), and HFNIHL% = 0.084 β + 0.147 (*R*^2^ = 0.938, *p* < 0.01, when CNE < 100 dB(A)∙year), respectively. The order of *R*^2^ was: *R*^2^_CNE_ > *R*^2^_ED_ > *R*^2^_kurtosis_. The results of nonlinear regression analyses showed that quadratic regressions for ED or CNE and sigmoid curve regression for kurtosis show a better fit than the other nonlinear models (Supplementary Table S3). The quadratic regression equations for ED and CNE were: HFNIHL% = 0.263 + 0.022 ED + 0.012 ED^2^ (*R*^2^’_ED_ = 0.985, *p* < 0.05), HFNIHL% = 0.048 + 0.092 CNE + 0.019 CNE^2^ (*R*^2^’_CNE_ = 0.988, *p* < 0.05), respectively. The sigmoid curve regression equation for kurtosis was: HFNIHL% = exp. (−0.426–1.202/β) (*R*^2^’_kurtosis_ = 0.968, *p* < 0.05, when CNE < 100 dB(A)∙year). The order of *R*^2^’ was: *R*^2^’_CNE_ > *R*^2^’_ED_ > *R*^2^’_kurtosis_.

**Table 2:** Relationship between prevalence of HFNIHL and noise exposure characteristics using the trend chi-squared test.

Noise metrics	Group	NIPTS_346_ (dB HL)	HFNIHL (%)	*χ* ^2^	*p* for trend
L_Aeq,8h_, dB(A)	<75	19.50 ± 5.85	0.00	21.85	<0.001
	75~	23.35 ± 12.45	34.22		
	85~	26.76 ± 14.49	40.04		
	95~	27.77 ± 15.43	47.02		
	105~	33.93 ± 14.52	66.10		
	Total	26.66 ± 14.43	41.64		
Exposure duration (ED), year	≤1	23.33 ± 15.13	30.72	52.56	<0.001
	1~	24.69 ± 13.02	32.84		
	5~	28.57 ± 14.78	46.03		
	10~	28.98 ± 14.36	54.17		
	15~	33.00 ± 14.53	67.29		
	Total	26.66 ± 14.43	41.64		
Kurtosis (CNE < 100, *n* = 624)	<10	18.59 ± 9.13	20.00	7.46	<0.01
	10~	24.73 ± 12.88	34.68		
	50~	26.58 ± 16.16	43.37		
	100~	31.78 ± 16.79	44.44		
	200~	40.71 ± 26.87	57.14		
	Total	25.19 ± 13.98	35.90		
CNE, dB(A)∙year	<80	23.15 ± 15.68	13.64	50.08	<0.001
	80~	25.26 ± 13.92	36.71		
	100~	28.06 ± 14.74	46.00		
	110~	33.45 ± 14.23	71.59		
	120~	48.53 ± 10.63	100.00		
	Total	26.66 ± 14.43	41.64		
Adj-CNE, dB(A)∙year	<80	17.68 ± 4.21	0.00	80.44	<0.001
	80~	23.98 ± 13.15	33.87		
	100~	26.49 ± 14.47	38.11		
	110~	34.64 ± 14.85	72.66		
	120~	46.68 ± 9.96	100		
	Total	26.66 ± 14.43	41.64		

NIPTS_346_, noise-induced permanent threshold shift at 3, 4, and 6 kHz frequencies; HFNIHL, high-frequency noise-induced hearing loss.

**Figure 3:**
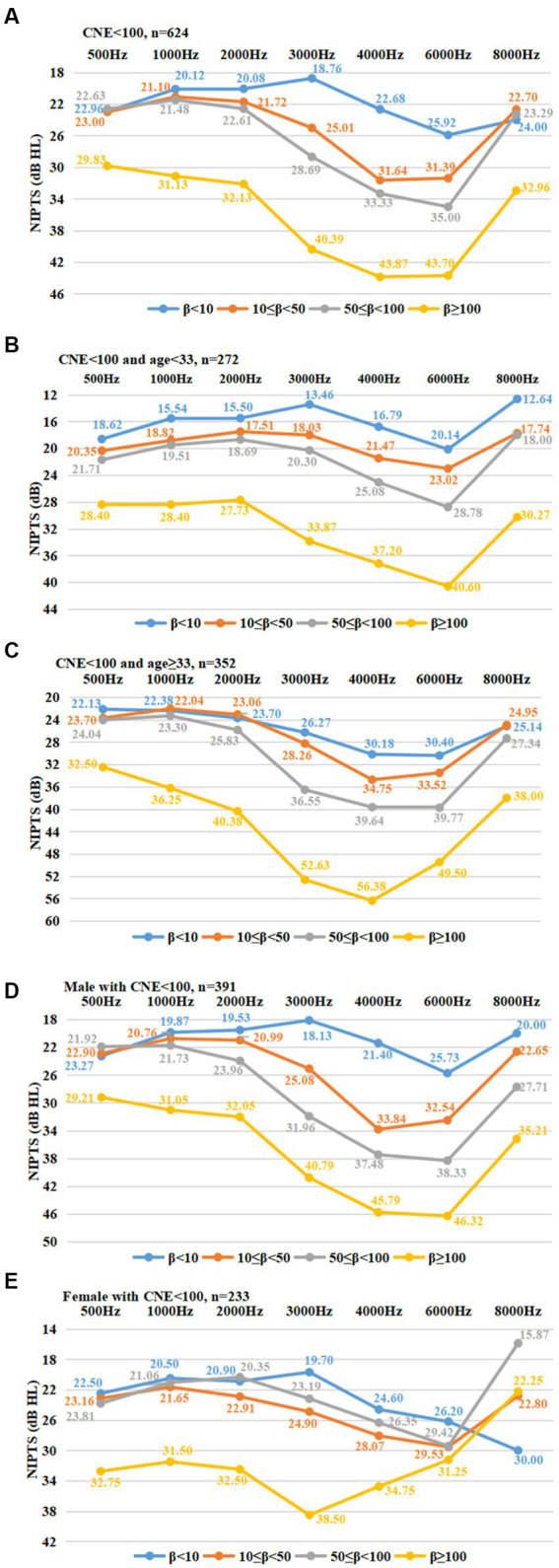
The notch at the high frequencies in NIPTS associated with kurtosis. **(A)**: A “V” shape notching phenomenon in NIPTS among workers with CNE < 100 dB(A)∙year. The notching extent became deeper with increasing kurtosis value, and reached the notching bottom at the frequency of 4 or 6 kHz; **(B)**: A “V” shape notching phenomenon in NIPTS among workers with CNE < 100 dB(A)∙year and age < 33 years. The notching extent became deeper with increasing kurtosis value, and reached the notching bottom at the frequency of 6 kHz; **(C)**: A “V” shape notching phenomenon in NIPTS among workers with CNE < 100 dB(A)∙year and age ≥ 33 years. The notching extent became deeper with increasing kurtosis value, and reached the notching bottom at the frequency of 4 or 6 kHz; **(D)**: A “V” shape notching phenomenon in NIPTS among male workers with CNE < 100 dB(A)∙year. The notching extent became deeper with increasing kurtosis value, and reached the notching bottom at the frequency of 4 or 6 kHz; **(E)**: There was no the “V” shape notching phenomenon in NIPTS among female workers with CNE < 100 dB(A)∙year. NIPTS: noise-induced permanent threshold shift at each frequency.

Figure 3A demonstrates that the mean NIPTS among 624 manufacturing workers with CNE < 100 dB(A)∙year gradually increased with frequencies from 0.5 kHz to 4 or 6 kHz, and gradually recovered at 8 kHz. The mean NIPTS curves exhibited a typical “V” shape notch across different frequencies. The notching extent became deeper with increasing kurtosis value and reached the notching bottom at the frequency of 4 or 6 kHz. Furthermore, Figures 3B–E show the notching phenomenon in NITPS stratified by age and sex among workers with CNE < 100 dB(A)∙year. The notching extent became deeper with the increase of kurtosis value and reached the notching bottom at the frequency of 4 or 6 kHz among workers in different age groups or male workers (Figures 3B–D). The phenomenon among female workers was not evident (Figure 3E).

In Table 3, the role of kurtosis in NIHL% at each frequency was evaluated using logistic regression analysis. The results demonstrated that with adjustment for age, sex, and CNE, there was a significant difference in the risk of NIHL between workers exposed to complex noise (*β* ≥ 10) and Gaussian noise (*β* < 10) at frequencies of 3, 4, 6, and 8 kHz (*p* < 0.05). The workers exposed to complex noise had a higher risk of NIHL than those exposed to Gaussian noise (OR > 2, *p* < 0.05). No significant differences were found in the NIHL% between complex noise exposure workers and Gaussian noise exposure workers at 0.5, 1, and 2 kHz (*p* > 0.05).

**Table 3:** The role of kurtosis in NIHL% at each frequency using logistic regression analysis.

Frequency (kHz)	Model 1			Model 2		
	OR (95%CI)		*P*	OR (95%CI)		*P*
0.5	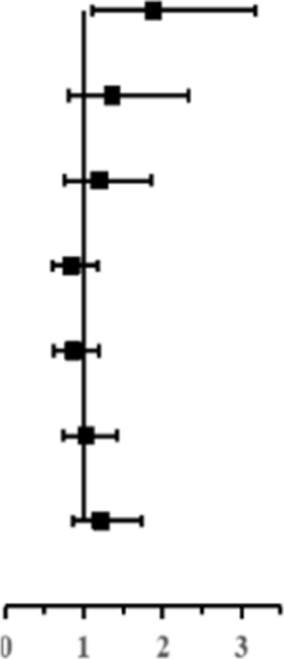	1.88 (1.11–3.18)	0.019	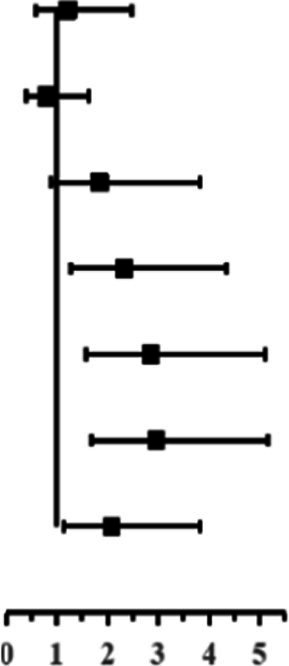	1.21 (0.59–2.47)	0.603
1		1.36 (0.80–2.33)	0.259		0.79 (0.39–1.61)	0.520
2		1.19 (0.76–1.85)	0.448		1.84 (0.89–3.81)	0.100
3		0.84 (0.60–1.17)	0.304		2.33 (1.26–4.34)	0.007
4		0.86 (0.62–1.19)	0.362		2.84 (1.58–5.11)	0.001
6		1.02 (0.74–1.41)	0.903		2.95 (1.69–5.16)	0.000
8		1.21 (0.85–1.74)	0.296		2.08 (1.13–3.83)	0.018

Model 1: Univariate logistic regression analysis (unadjusted), Model 2: Multivariate logistic regression analysis adjusted by age, sex, and CNE; NIHL% at each frequency as a categorical dependent variable.

Age (years): <33≥33; Sex: male, female; CNE [dB(A)∙year]: <80, 80~, 100~, 110~, 120~; Kurtosis:< 10, ≥10.

### The effectiveness of adj-CNE in evaluating occupational NIHL

**Figure 4:**
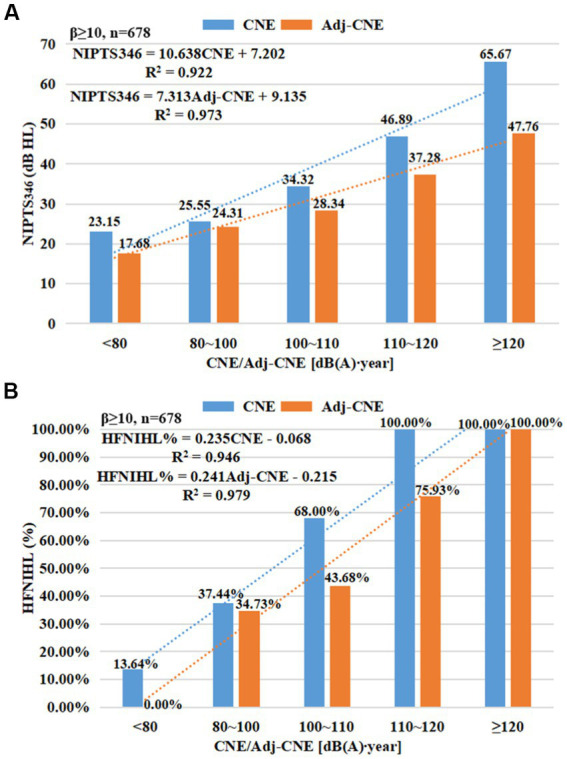
The linear relationships between NIHL and CNE before and after adjustment by kurtosis. **(A)**: an increase of *R*^2^ from 0.922 to 0.973 after CNE adjusted with kurtosis using NIPTS_346_ as targeted effect; **(B)**: an increase of *R*^2^ from 0.946 to 0.979 after CNE adjusted with kurtosis using HFNIHL% as targeted effect.

Table 1 shows that the mean adj-CNE among 867 manufacturing workers was 99.85 ± 10.20 dB(A)∙year. Table 2 shows that the mean NIPTS_346_ level and HFNIHL% were the highest in the group with adj-CNE greater than 120 dB(A)∙year (48.53 ± 10.63 dB HL). The group with adj-CNE less than 80 dB(A)∙year had the lowest NIPTS_346_ (23.15 ± 15.68 dB HL) and HFNIHL% (13.67%), and HFNIHL% increased with increasing adj-CNE (*p* < 0.001). Figure 4 illustrates a strong linear relationship between NIHL and CNE or adj-CNE when β ≥ 10. The two linear regression equations between NIPTS_346_ and CNE or adj-CNE were: NIPTS_346_ = 10.638 CNE + 7.202 (*R*^2^ = 0.922), and NIPTS_346_ = 7.313 adj-CNE + 9.135 (*R*^2^ = 0.973), respectively (Figure 4A). The two linear regression equations between HFNIHL% and CNE or adj-CNE were: HFNIHL% = 0.235 CNE-0.068 (*R*^2^ = 0.946), and HFNIHL% = 0.241 adj-CNE-0.215 (*R*^2^ = 0.979), respectively (Figure 4B). The *R*^2^ was increased after CNE was adjusted with kurtosis using NIPTS_346_ or HFNIHL% as the targeted effect. Table 4 demonstrates the calculated differences in NIPTS_346_ and HFNIHL% among Gaussian and complex noise-exposed workers before and after CNE adjustment by kurtosis. When mean NIPTS_346_ and HENIHL% were evaluated using unadjusted CNE, significant differences between the complex and Gaussian noise groups were observed (*p* < 0.05). However, after CNE was adjusted by kurtosis, these differences were diminished significantly (*p* < 0.05), i.e., the average difference in NIPTS_346_ decreased to 5.8 dB HL from 11.44 dB HL, and the average difference in HFNIHL% declined to 12.89% from 25.39% (*p* < 0.05).

**Table 4:** Differences in NIHL between complex noise and Gaussian noise before and after adjustment by kurtosis.

CNE/ Adj-CNE	NIPTS_346_ (Mean, dB HL)					CNE/ Adj-CNE	HFNIHL%				
	G	C		D1	D2		G	C		D3	D4
	CNE	CNE	Adj-CNE				CNE	CNE	Adj-CNE		
<90	15.00	22.69	22.56	7.69	7.56	<90	0.00	29.38	27.59	29.38	27.59
90~	23.90	26.75	24.69	2.85	0.79	90~	42.86	40.70	36.89	−2.16	−5.97
95~	16.61	27.21	24.81	10.60	8.20	95~	11.76	40.21	35.44	28.45	23.68
100~	20.81	32.39	28.09	11.58	7.28	100~	26.32	66.67	41.73	40.35	15.41
105~	22.48	38.41	28.85	15.93	6.37	105~	20.00	70.83	47.62	50.83	27.62
110~	30.29	34.83	33.54	4.54	3.25	110~	61.11	100.00	70.27	38.89	9.16
115~	36.02	52.92	45.42	16.90	9.40	115~	82.61	100.00	88.24	17.39	5.63
120~	44.25	65.67	47.76	21.42	3.51	120~	100.00	100.00	100.00	0.00	0.00
Average	26.17	37.61	31.97	11.44	5.80^a^	Average	43.08	68.47	55.97	25.39	12.89^b^

G, Gaussian noise; C, complex noise.

D1, Difference in NIPTS_346_ between complex noise exposure workers and Gaussian noise exposure workers after grouped by CNE; D2, Difference in NIPTS_346_ between complex noise exposure workers and Gaussian noise exposure workers after grouped by adj-CNE; D3, Difference in HFNIHL% between complex noise exposure workers and Gaussian noise exposure workers after grouped by CNE; D4, Difference in HFNIHL% between complex noise exposure workers and Gaussian noise exposure workers after grouped by adj-CNE.

a *p* < 0.05, compared with D1.

b *p* < 0.05, compared with D3.

## Discussion

This study focused on the role of noise kurtosis (an indirect indicator for noise’s temporal structure) and adj-CNE in occupational NIHL among metal manufacturing workers. The results showed that the effect of noise temporal structure on occupational NIHL associated with complex noise should not be ignored in the metal manufacturing industry. After complex noise’s CNE was adjusted by kurtosis, its dose–response relationship with NIHL was closer to that of Gaussian noise. It indicates that as an auxiliary metric, kurtosis combined with noise energy metrics is a new indicator that could effectively evaluate the risk of occupational NIHL.

Each type of work in the metal manufacturing industry had a specific noise exposure level (e.g., L_Aeq,8h_, CNE), kurtosis value, and NIHL%. High levels of L_Aeq,8h_ [88.02 ± 5.24 dB(A)] and noise’s temporal complexity (40.91 ± 51.22), with relatively high NIPTS_346_ (26.67 ± 14.43 dB HL) and HFNIHL% (40.41%) were found in metal manufacturing industries, which were consistent with previous studies (14, 24, 26). These studies reported that 62.53% of automotive manufacturing workers in China were exposed to noise levels that exceeded 85 dB(A), with HFNIHL% of 28.82, and 65.6% of metal manufacturing workers in China had HFNIHL, with a higher L_Aeq,8h_ level (95.25 ± 3 dB(A)). One systematic review on occupational workers from the oil field, electrolytic aluminum, and automobile industries found that the NIHL% in China was 21.3%, with HFNIHL of 30.2% and L_Aeq,8h_ level of 98.4 ± 7.2 dB(A) (29). Another showed that 23,261 workers from manufacturing and mining industries, including forging, riveting, stamping, casting, drilling, molding, finishing, pressing, assembling, welding, grinding, smashing, steel rolling, wood sawing, and machine testing, were exposed to 88.7 ± 6.9 dB(A) noise levels, with a higher kurtosis value of 40.3 ± 79.5 and HFNIHL% of 34.2% (30).

HFNIHL% increased with increasing ED, L_Aeq,8h_, CNE, or kurtosis. Strong linear and nonlinear relationships between HFNIHL% and ED, CNE, or kurtosis (when CNE < 100 dB(A)∙year) were observed, and the order of their *R*^2^ was: *R*^2^_CNE_ > *R*^2^_ED_ > *R*^2^_kurtosis_. A study on 2,333 manufacturing workers from 34 industries in China showed that HFNIHL% increased with increasing kurtosis values and L_Aeq,8h_, and the *R*^2^ values of linear regression equations between kurtosis or L_Aeq,8h_ and HFNIHL% were 0.911(when CNE < 100 dB(A)∙year) and 0.988, respectively (19). These studies indicate that noise energy metrics contributed the most to NIHL, followed by kurtosis. Furthermore, Figure 1 shows no correlation between kurtosis and L_Aeq,8h_, which suggests that noise energy and temporal structure were independent factors and might be combined into one index. In this study, the effect of kurtosis on mean NIPTS across different frequencies was further explored among metal manufacturing workers at not very high noise energy levels (e.g., CNE < 100 dB(A)∙year). The typical ‘V’ shape notching extent in mean NIPTS became deeper with an increase in kurtosis value; It reached the notching bottom at the frequency of 4 or 6 kHz among workers in different age groups or male workers, which may be explained by the pathways that greater NIPTS and outer hair cell damage were observed at 4 kHz octave band of noise than those at 0.5 kHz octave band of noise among chinchilla (31), who has a similar auditory system to humans (32). The possible reason for the ‘V’ shape notching only in males may be that the male workers suffered more significant HFNIHL% caused by complex noise than females (33), and they were more likely exposed to complex noise environments such as punching, stamping, metalworking, woodworking, and nail gunning (34). Besides, one study of 1,404 Chinese manufacturing workers reported similar findings that the V- or U-shaped notch phenotypes of NIPTS broadens and deepens with increasing kurtosis, even widened to 1–8 kHz when β > 100 (27). Notably, the kurtosis-associated notch initially occurred at high frequencies and reached the notching bottom at 4 or 6 kHz, with the notch range of 3–8 kHz when β < 10, indicating that noise exposure led to greater hearing loss at high frequencies. A highly complex temporal structure aggravated the development of NIHL at high frequencies. Another study of 1962 Chinese manufacturing workers (CNE < 100 dB(A)∙year) found that the average NIPTS increased gradually with frequencies from 0.5 kHz to 4 kHz and gradually decreased at 8 kHz, and the notching extent in NIPTS at the high frequencies 3, 4, and 6 kHz became deeper with kurtosis and reached the notching bottom at 4 kHz (19). These results supported the present study.

Moreover, logistic regression analysis suggested that after adjusting for age, sex, and CNE, kurtosis greater than 10 led to a higher risk for NIHL than that less than 10 since the OR value was greater than 2 at the frequency of 3, 4, 6, or 8 kHz, which suggested that complex noise was more harmful to the auditory system than Gaussian noise; There was an independent effect of kurtosis on NIHL development after controlling for CNE and age. These results agreed with previous studies (15, 17, 24, 27). For example, Zhao et al. found that workers exposed to complex noise had a higher risk of NIHL than those exposed to Gaussian noise (OR = 1.806) (24). A meta-analysis of 71, 865 Chinese manufacturing workers suggested that complex noise contributed to greater HFNIHL% than Gaussian noise (OR = 1.95) (29). Shi et al. also reported that workers exposed to complex noise were at a higher risk of HFNIHL than Gaussian noise-exposed workers (OR = 2.20) (30). A number of animal experiments observed that using only noise energy metrics for assessing NIHL caused by complex noise may underestimate the extent of hearing trauma (20, 23, 35, 36). Several human investigations confirmed these animal test results (13–15, 37). Moreover, the kurtosis-associated risks of NIHL at high frequencies of 3, 4, 6, and 8 kHz were in line with the findings that greater hearing loss (75 dB) caused by noise exposure alone was observed at high frequencies than that at lower frequencies (40 dB) (38). These findings indicate that CNE, as a comprehensive metric comprising L_Aeq,8h_ and exposure duration, was a major but not a unique determinant for NIHL. The effect of noise temporal structure on NIHL should not be ignored.

Considering the contribution of energy and kurtosis to NIHL, some correction methods, in which kurtosis was used to adjust noise energy, have been developed, such as the adjustment method against exposure duration of noise and the correction method against L_Aeq,8h_ (14, 39). This study used the correction method that uses kurtosis to adjust exposure duration. The adj-CNE was a new index for quantifying complex noise exposure. The forging, grinding, or polishing workers with the highest adj-CNE level (when β ≥ 10) had the highest NIPTS_346_ and HFNIHL%. NIPTS_346_ and HFNIHL% increased with increasing adj-CNE, and a better dose–response relationship for complex noise exposure was observed between adj-CNE and HFNIHL% or NIPTS_346_ than between CNE and HFNIHL% or NIPTS_346_. Table 4 illustrates that the average difference in NIPTS_346_ or HFNIHL% between complex noise and Gaussian noise for adj-CNE was significantly reduced as compared with CNE, indicating that the dose–response relationship between adj-CNE and NIHL associated with complex noise became close to the dose–response relationship of Gaussian noise (served as a baseline). These findings were supported by previous studies (14, 15, 21, 22). A study among 341 steel and textile manufacturing workers found that adj-CNE improved the correlation with NIHL and provided a single metric for dose–response effects across different types of noise (15). Zhao et al. recruited 195 workers from a textile manufacturing plant and a metal fabrication facility in China. They found that the two dose–response lines between the NIHL% and adj-CNE for complex noise almost overlapped with those for Gaussian noise (14). A study on 2,898 manufacturing workers from Zhejiang Province, China, indicated that after CNE was adjusted by kurtosis, the difference in average HFNIHL% between complex and Gaussian noise was significantly reduced from 7.40 to 1.28%, and the two regression curves nearly overlapped (21). The 3,102 Chinese manufacturing workers also supported this point (22). These results suggest that adj-CNE, a comprehensive metric combining noise energy and temporal structure, can assess the risk of occupational NIHL across different types of noise more effectively than CNE alone.

Two limitations merit consideration in this study. One limitation is that the typical ‘V’ shape notching phenomenon among female workers was not found. The reason may be that the number of females (*n* = 233) among the enrolled 867 manufacturing workers was too small. Another is that we judged the effectiveness of adj-CNE using only the dose–response relationship between adj-CNE and NIPTS_346_ or HFNIHL%. It is necessary to develop multiple methods to confirm the role of adj-CNE in epidemiological investigations with larger sample sizes.

## Conclusion

Besides noise energy, noise kurtosis was a key factor influencing occupational NIHL among metal manufacturing workers. After complex noise’s CNE was adjusted by kurtosis, its dose–response relationship with NIHL was closer to that of Gaussian noise, indicating that adj-CNE could assess the risk of NIHL more effectively than CNE alone. More epidemiological investigations with a wide range of specific manufacturing industries are needed to confirm the validity of the adj-CNE metric for assessing NIHL.

## Data availability statement

The raw data supporting the conclusions of this article will be made available by the authors, without undue reservation.

## Ethics statement

The studies involving human participants were reviewed and approved by the Ethics Committee of the Zhejiang Center for Disease Control and Prevention, China (ZJCDC-T-043-R). The patients/participants provided their written informed consent to participate in this study.

## Author contributions

SYL conducted the statistical analysis and wrote the manuscript. SL oversaw data analysis and interpretation. JRX, YL, and PYQ checked data and the results of analysis. JRX and YQC carried out the experiment. MBZ was responsible for data collection and the final manuscript. MZ was responsible for data collection and the final manuscript. All authors approved the final manuscript.

## Funding

This work was supported by the Pre-research project on occupational health standards (20210102); the National Institutes of Health, National Institute on Deafness and Other Communication Disorders, United States (1R01DC015990); and the Fundamental Research Funds for the Central University (YCJJ202201031).

## Conflict of interest

The authors declare that the research was conducted in the absence of any commercial or financial relationships that could be construed as a potential conflict of interest.

## Publisher’s note

All claims expressed in this article are solely those of the authors and do not necessarily represent those of their affiliated organizations, or those of the publisher, the editors and the reviewers. Any product that may be evaluated in this article, or claim that may be made by its manufacturer, is not guaranteed or endorsed by the publisher.
