# Supplementary material for: The role of kurtosis and kurtosis-adjusted energy metric in occupational noise-induced hearing loss among metal manufacturing workers
Source: Front Public Health. 2023 Jun 29;11:1159348. doi: 10.3389/fpubh.2023.1159348 (PMC10344449; doi:10.3389/fpubh.2023.1159348)
Supplement: Supplementary file 1 [file Table_1.docx]

***Supplementary Material***

**The role of kurtosis and kurtosis-adjusted energy metric in occupational noise-induced hearing loss among metal manufacturing workers**

**Supplementary Tables**

| **Table S1. Inclusion criteria for the subjects.** | |
| --- | --- |
| **Number** | **Inclusion criteria** |
| 1 | Never worked in high noise-level environments from different enterprises |
| 2 | No history of military service or shooting activity |
| 3 | No chief complaint of hearing loss, and no auditory symptoms |
| 4 | No family history of hearing loss |
| 5 | No history of ear disease, taking ototoxic drugs, and diabetes |
| 6 | No or minimal use of a hearing protection device (HPD) |
| 7 | No co-exposure to noise and ototoxic drugs |

| **Table S2. The collected data through questionnaire for the present study.** | |
| --- | --- |
| **Content** | **Data** |
| General information | Age |
|  | Sex |
|  | Shooting activity |
|  | Military service |
|  | Family history of hearing loss |
| Occupational history | Industry |
|  | Workshop |
|  | Type of work |
|  | Noise exposure duration (ED) |
|  | Hearing protection device (HPD) use |
|  | Worked in high noise-level environment before |
|  | Co-exposure to noise and ototoxic drugs |
| Overall health level | Ear disease history |
|  | Ototoxic drug intake |
|  | Diabetes |
|  | Chief complaint of hearing loss, |
|  | Auditory symptoms |

| **Table S3. The nonlinear regression between exposure metrics and HFNIHL%.** | | | | | | | | |
| --- | --- | --- | --- | --- | --- | --- | --- | --- |
| Model | Metrics | R^2^ | F | *P* | Constants | B_1_ | B_2_ | B_3_ |
| Logarithmic | ED | 0.842 | 15.972 | <0.05 | 0.252 | 0.220 | - | - |
|  | CNE | 0.888 | 23.901 | <0.05 | 0.064 | 0.493 | - | - |
|  | Kurtosis | 0.956 | 65.641 | <0.01 | 0.197 | 0.211 | - | - |
| Inverse | ED | 0.659 | 5.795 | >0.05 | 0.636 | -0.381 | - | - |
|  | CNE | 0.742 | 8.619 | >0.05 | 0.939 | -0.882 | - | - |
|  | Kurtosis | 0.894 | 25.182 | <0.05 | 0.582 | -0.400 | - | - |
| Quadratic | ED | 0.985 | 67.740 | <0.05 | 0.263 | 0.022 | 0.012 | - |
|  | CNE | 0.988 | 79.794 | <0.05 | 0.048 | 0.092 | 0.019 | - |
|  | Kurtosis | 0.951 | 19.205 | <0.05 | 0.089 | 0.134 | -0.008 | - |
| Cubic | ED | 0.989 | 31.279 | >0.05 | 0.349 | -0.098 | 0.058 | -0.005 |
|  | CNE | 0.994 | 53.976 | >0.05 | -0.184 | 0.418 | -0.105 | 0.014 |
|  | Kurtosis | 0.992 | 39.974 | >0.05 | -0.157 | 0.480 | -0.140 | 0.015 |
| Composite | ED | 0.972 | 104.133 | <0.05 | 0.238 | 1.230 | - | - |
|  | CNE | 0.934 | 42.416 | <0.05 | 0.109 | 1.592 | - | - |
|  | Kurtosis | 0.877 | 21.371 | <0.05 | 0.187 | 1.265 | - | - |
| Power | ED | 0.886 | 23.312 | <0.05 | 0.276 | 0.491 | - | - |
|  | CNE | 0.984 | 186.704 | <0.05 | 0.141 | 1.188 | - | - |
|  | Kurtosis | 0.966 | 85.978 | <0.05 | 0.210 | 0.613 | - | - |
| Sigmoid curve | ED | 0.721 | 7.743 | >0.05 | -0.419 | -0.868 | - | - |
|  | CNE | 0.947 | 53.416 | <0.05 | 0.222 | 0.465 | - | - |
|  | Kurtosis | 0.968 | 91.348 | <0.05 | -0.426 | -1.202 | - | - |
| Growth | ED | 0.972 | 104.133 | <0.05 | -1.436 | 0.207 | - | - |
|  | CNE | 0.934 | 42.416 | <0.05 | -2.217 | 0.465 | - | - |
|  | Kurtosis | 0.877 | 21.371 | <0.05 | -1.679 | 0.235 | - | - |
| Exponential | ED | 0.972 | 104.133 | <0.05 | 0.238 | 0.207 | - | - |
|  | CNE | 0.934 | 42.416 | <0.05 | 0.109 | 0.465 | - | - |
|  | Kurtosis | 0.877 | 21.371 | <0.05 | 0.187 | 0.235 | - | - |

R^2^: Coefficient of determination; B: Coefficients of parameter estimation.
